# Changes in leg cycling muscle synergies after training augmented by functional electrical stimulation in subacute stroke survivors: a pilot study

**DOI:** 10.1186/s12984-020-00662-w

**Published:** 2020-02-27

**Authors:** Emilia Ambrosini, Monica Parati, Elisabetta Peri, Cristiano De Marchis, Claudia Nava, Alessandra Pedrocchi, Giorgio Ferriero, Simona Ferrante

**Affiliations:** 1 Department of Electronics, Information and Bioengineering, Neuroengineering and Medical Robotics Laboratory, Politecnico di Milano, Milan, Italy; 2 Istituti Clinici Scientifici Maugeri, IRCSS, Lissone, Italy; 3 Department of Electrical Engineering, Laboratory of Biomedical Diagnostics, Eindhoven University of Technology, Eindhoven, The Netherlands; 4 Department of Engineering, Roma Tre University, Rome, Italy

**Keywords:** Stroke, Rehabilitation, Functional electrical stimulation, Cycling, Lower limb, Electromyography, Muscle synergy

## Abstract

**Background:**

Muscle synergies analysis can provide a deep understanding of motor impairment after stroke and of changes after rehabilitation. In this study, the neuro-mechanical analysis of leg cycling was used to longitudinally investigate the motor recovery process coupled with cycling training augmented by Functional Electrical Stimulation (FES) in subacute stroke survivors.

**Methods:**

Subjects with ischemic subacute stroke participated in a 3-week training of FES-cycling with visual biofeedback plus usual care. Participants were evaluated before and after the intervention through clinical scales, gait spatio-temporal parameters derived from an instrumented mat, and a voluntary pedaling test. Biomechanical metrics (work produced by the two legs, mechanical effectiveness and symmetry indexes) and bilateral electromyography from 9 leg muscles were acquired during the voluntary pedaling test. To extract muscles synergies, the Weighted Nonnegative Matrix Factorization algorithm was applied to the normalized EMG envelopes. Synergy complexity was measured by the number of synergies required to explain more than 90% of the total variance of the normalized EMG envelopes and variance accounted for by one synergy. Regardless the inter-subject differences in the number of extracted synergies, 4 synergies were extracted from each patient and the cosine-similarity between patients and healthy weight vectors was computed.

**Results:**

Nine patients (median age of 75 years and median time post-stroke of 2 weeks) were recruited. Significant improvements in terms of clinical scales, gait parameters and work produced by the affected leg were obtained after training. Synergy complexity well correlated to the level of motor impairment at baseline, but it did not change after training. We found a significant improvement in the similarity of the synergy responsible of the knee flexion during the pulling phase of the pedaling cycle, which was the mostly compromised at baseline. This improvement may indicate the re-learning of a more physiological motor strategy.

**Conclusions:**

Our findings support the use of the neuro-mechanical analysis of cycling as a method to assess motor recovery after stroke, mainly in an early phase, when gait evaluation is not yet possible. The improvement in the modular coordination of pedaling correlated with the improvement in motor functions and walking ability achieved at the end of the intervention support the role of FES-cycling in enhancing motor re-learning after stroke but need to be confirmed in a controlled study with a larger sample size.

**Trial registration:**

ClinicalTrial.gov, NCT02439515. Registered on May 8, 2015, .

## Background

The loss of lower limb strength and motor coordination are the most common deficits causing long term disability after stroke [1, 2]. Individuals with stroke suffer from muscle weakness, inappropriate muscle activity and abnormal intermuscular coordination, which are the major factors contributing to asymmetrical movements and alteration of locomotor abilities [3].

Within the current stroke rehabilitation practice, motor impairments and its recovery are predominantly measured through clinical scales, which provide standardized, comprehensive and easy to use evaluations for clinical practitioners [4]. Even though these tools are the gold standard in the characterization of patients’ motor impairment, they do not provide quantitative data to offer insights into the motor recovery process [5]. Complementary to those assessments, instrumental techniques tailored to quantitatively examine the neuro-mechanics of task-specific exercises, such as walking [6, 7], balance [8, 9] or cycling [10, 11], could represent a step forward towards the understanding of patient’s condition as well as the development of customized neuro-rehabilitative interventions which might enhance motor re-learning after stroke [12]. In this context, multi-muscle activity recorded through surface electromyography (EMG) and quantitatively analyzed in terms of muscle coordination represents a promising powerful tool to examine motor impairments [13]. The hypothesis of this approach is that the muscle recruitment process involved in the planning and execution of complex movements is simplified in a low-dimensional organization of invariant spatio-temporal modules, commonly termed muscle synergies [14]. A muscle synergy is a functional group of muscles simultaneously contracting during a motor task [13]. Muscle synergy analysis has been applied both in healthy controls and neurological patients for different motor tasks, especially for walking [6, 15, 16].

When muscle synergy analysis has been applied on stroke survivors, several studies observed a reduction in the number of independent motor modules compared to healthy controls, negatively correlated with the level of motor impairment [6, 11, 17]. Abnormal co-activation patterns in the paretic upper limb of stroke patients, resulting in a reduced number of possible muscle combinations, were already observed in 1995 [18]. The reduced number of independent motor modules was mainly explained by a merging of the healthy motor modules, consistent with a decrease in the independence of the corticospinal drive [6, 12]. However, in a different study [19], which investigated the modular coordination during locomotion of a group of subacute stroke patients, a similar number of motor modules was found between patients and controls but it was not possible to accurately describe the muscular activation pattern of subacute stroke patients using the same motor modules of healthy controls.

Few studies explored the changes of muscle synergies after a rehabilitative intervention [20–25]. Two of them were focused on upper limb motor recovery in chronic stroke patients [20, 21]. One study investigated muscle synergies changes during walking in children with Cerebral Palsy [22]. The remaining three studies observed longitudinal changes in stroke survivors during locomotion [23–25]. Routson and colleagues [23] observed that 12 weeks of treadmill training with body weight support were able to influence module composition and timing. Indeed, a better timing of the ankle plantar flexor module and an increased number of modules were found which overall improved the walking performance of chronic stroke patients. Conversely, in another study evaluating longitudinal changes in modular motor coordination during locomotion in subacute stroke survivors, no substantial changes in the number of motor synergies were found after 1 month of conventional therapy [24]. Finally, in the most recent study on subacute stroke survivors an increased lateral symmetry in muscle synergies while walking, associated with improvements in gait kinematics measurements, was found after 3 weeks of walking training supported by a lower limb exoskeleton [25]. Overall, it remains uncertain whether a rehabilitation treatment can alter motor coordination as measured by muscle synergies and how much this alteration is associated with motor recovery after stroke.

In the context of lower-limb rehabilitation, a repetitive, intensive and task-oriented intervention is represented by leg-cycling training augmented by Functional Electrical Stimulation (termed as FES-cycling). FES-cycling training achieved promising results over time in the subacute phase after stroke, improving locomotion ability [26–28], strength of lower limb muscles [27] and motor coordination [26]. Since it does not require the capability to maintain standing balance, FES-cycling can be one of the best training options for patients initially not able to walk [28]; for the same reason, leg-cycling can be used as a quantitative assessment technique [5, 29].

Muscle synergies during cycling have been analyzed in previous researches on healthy subjects, revealing a complexity of three [10, 30] or four motor modules [11, 31]. Only one study compared muscle synergies of healthy elderly subjects with respect to stroke patients during recumbent cycling [11]. Four muscle synergies were identified for the healthy group: Synergy 1 describes the knee extensor activity responsible for power production; Synergy 2 mainly reflects the activity of hamstrings, ankle plantar-flexors and gluteus maximus during the transition from extension to flexion; Synergy 3 provides the knee flexor activity during limb recovery; and Synergy 4 combined the activities of rectus femoris, tensor fascia latae and ankle dorsiflexors for assisting leg transfer between flexion and extension. A reduced number of synergies was extracted for the affected leg of the most impaired patients, therefore confirming that the fewer modules resulted from a merging of the healthy synergy modules, as seen in previous studies during walking [6].

Muscle synergy analysis during cycling has never been used to investigate the motor recovery process after stroke coupled with a rehabilitative intervention. Therefore, the purpose of the present study was to evaluate the longitudinal changes in the modular motor coordination during leg-cycling in a group of subacute stroke patients involved in a 3-week intervention of FES-augmented cycling. Furthermore, we investigated the relationship between these changes and changes in motor function and locomotion ability.

## Materials and methods

### Participants

Participants were a subgroup of patients selected from a larger clinical trial carried out at IRCCS Istituti Clinici Scientifici Maugeri, Lissone, Monza Brianza (MB), Italy (Clinical trial Identifier: NCT02439515). This clinical trial was aimed at investigating the effects of a multimodal biofeedback training, including FES-cycling, after stroke [27, 28]. Inclusion criteria were: first ever stroke within 6 months; hemiparesis secondary to a single unilateral stroke; low spasticity at the lower limbs (Modified Ashworth Scale < 2); ability to tolerate FES. Exclusion criteria were: allergy to stimulation electrodes; limitation at joint mobility; cognitive impairment (Mini Mental State Evaluation < 20); spatial hemineglect; other neurological comorbidities and presence of cardiac pacemakers.

Reference data obtained in a previous study [11] on a group of 12 healthy subjects (8 males, 4 females, mean age of 68 ± 5 years) were used for comparisons

The Ethical committee of the rehabilitation center approved the study (date of approval: 10/03/2014) and all subjects provided a written informed consent.

### Intervention

**Fig. 1:**
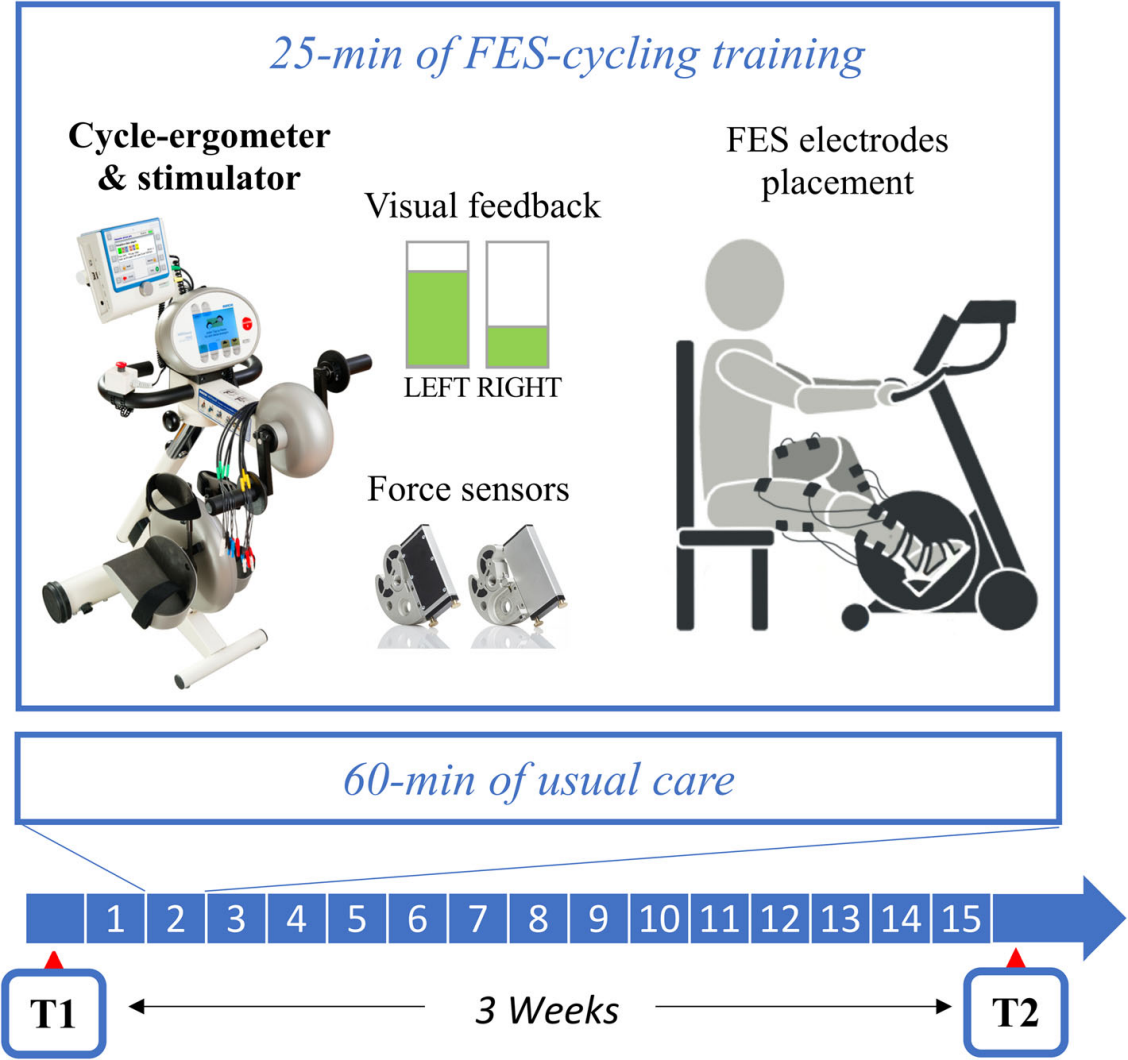
Experimental setup used for FES-cycling training. The intervention lasted 3 weeks (15 sessions) and each daily session consisted of 60 min of usual care and 25 min of FES-cycling training. FES-cycling required the use of a cycle-ergometer, force sensors at the pedals, and a neuromuscular electrical stimulator connected to 8 muscles (4 for each legs). The figure shows also the visual feedback displayed to the subject during training and the FES electrodes placement (on the right)

Participants underwent a neuro-rehabilitative in-patient intervention for 3 weeks, 5 times per week. Each session consisted of 60 min of usual care and 25 min of voluntary cycling on a motorized cycle-ergometer (MOTOmed Viva2 ergometer, Reck GmbH, Germany) augmented by FES. The ergometer was equipped with force sensors (PowerForce™, Radlabor GmbH), which measured the radial and tangential forces at the two pedals. A visual feedback of the tangential forces produced by the two legs was provided to promote the execution of a symmetrical task [27, 28]. An 8-channel current-controlled stimulator (RehaMove2™ Hasomed GmbH, Germany) coordinated the bilateral neuromuscular stimulation of the quadriceps, hamstrings, tibialis anterior and gastrocnemius lateralis muscles, according to a biomimetic stimulation strategy, defined on the basis of physiological muscle activation strategy of a group of young healthy subjects while voluntarily pedaling on the same ergometer [32]. Self-adhesive bipolar surface electrodes (Pals® electrodes, Axelgaard Manufacturing Co., Ltd.), applied proximally and distally to the motor point of the muscles, delivered rectangular biphasic pulses with a pulse width of 400 μs and a stimulation frequency of 20 Hz. Current amplitude on each muscle was customized for each patient and could vary among participants in accordance to individual comfort. The current amplitudes delivered to the affected leg was chosen to produce a visible tolerated functional muscle contraction, while the unaffected leg received a stimulation intensity just above the sensory threshold. Pedaling resistance was set at a comfortable level that still permitted participants to smoothly cycle for the entire training session. Figure 1 shows the experimental setup used for training, the FES electrodes placement, as well as the intervention schedule.

### Assessment

Participants were tested through clinical scales, gait analysis and neuro-mechanical evaluation of voluntary pedaling before (T1) and after (T2) the end of the intervention.

The lower limb component of the Motricity Index (range: 0–100) was used to grade the muscle power of the affected lower limb. The Berg Balance Scale (BBS) and the motor subscale of the Functional Independent Measure (FIM mot) were adopted to determine the functional mobility (range: 0–56) and the required degree of assistance (range: 13–91), respectively. Higher values result in lower level of impairment.

Gait analysis was performed using the GaitRite mat (CIR System Inc. USA) to assess spatial and temporal gait parameters: gait speed (cm/s), step length (cm), step time (s), double support time (s), swing velocity (cm/s). Each participant completed 3 walking trials at comfortable speed and the results were averaged across trials.

All patients underwent a neuro-mechanical assessment of voluntary pedaling using the same sensorized cycle-ergometer used for training. The test consisted of four trials at different cadences, performed in a random order (20, 30, 40, 50 revolution per minute, RPM). Each trial started with 1 minute of passive cycling in which the ergometer’s motor maintained a constant cadence without any voluntary contribution of the patient (passive phase); then the cadence maintained by the motor was reduced by 10 RPM and the patient was asked to voluntarily pedal for 2 min maintaining the same target cadence of the passive phase (active phase) [11]. It was chosen to perform isokinetic trials to have more repeatable pedaling cycles in terms of forces and EMG activations. The motor was kept ON also during the active phase in order to guarantee a smooth pedaling movement; however, being the cadence maintained by the motor lower than the target cadence, the voluntary involvement of the patient was assured. A minimum wash-out period of 5 min was guaranteed between consecutive trials to prevent muscle fatigue.

During the trials, the crank angle and the tangential and radial forces at the two pedals were acquired with a sampling frequency of 1000 Hz. Surface EMG signals of 9 muscles of both lower limbs **(**Gluteus Maximus (Gmax), Biceps Femoris long head (BFlh), Biceps Femoris short head (BFsh), Gastrocnemius Medialis (GAS), Soleus (SOL), Tensor Fasciae Latae (TFL), Rectus Femoris (RF), Vastus Lateralis (VL), and Tibialis Anterior (TA)) were acquired by a multi-channel signal amplifier (Porti 32™, TMS International) and sampled at 1024 Hz. The position of the self-adhesive Ag/AgCl electrodes (Kendall™, COVIDIEN) and the preparation of the skin followed the Surface Electromyography for Non-Invasive Assessment of Muscles (SENIAM) recommendations [33].

### Data processing

All signals recorded during the neuro-mechanical assessment were processed and synchronized using a customized MATLAB program (R2018b, MathWorks Inc., Natick, MA, USA). Trials at different cadences were analyzed separately.

#### Muscle synergies analysis

The raw EMG signals were firstly filtered with a 3rd order band-pass (20-400 Hz) Butterworth filter. Then, the filtered signals were full-wave rectified and low-pass filtered at 5 Hz to obtain the linear EMG envelope. These envelopes were segmented by pedaling cycle using the synchronized crank angle and only cycles of the active phase which matched the trial target cadence with a tolerable range of ± 4 RPM were considered for the subsequent analysis. The segmented EMG envelopes were resampled in 360-points vectors with a cubic spline approximation and the left-side profiles were shifted by 180° [11]. Finally, the EMG envelopes were normalized to the median peak value calculated across pedaling cycles within each trial.

The Weighted Nonnegative Matrix Factorization algorithm (WNMF) was used to extract muscle synergies [22]. The WNMF algorithm was selected to adjust the analysis in the cases in which some muscles were missing (mostly occurring at Gmax). Indeed, a weight of 1 was assigned to acquired muscles and of 0 to non-acquired muscles. The algorithm used a multiplicative update rules to iteratively decompose the EMG envelopes in which the minimum threshold for convergence was set at 1 × 10^− 5^ for at least 30 repetitions and the maximum number of repetitions was fixed at 1 × 10^3^. The WNMF was applied 30 times on 30 different datasets in order to ensure extraction consistency. Each dataset was composed by the normalized EMG envelopes of 30 pedaling cycles randomly selected among the available ones. Therefore, the WNMF was applied on a matrix *M*_*(9 × 10800)*_ where 9 are the recorded muscles and 10,800 are the total number of EMG samples used in the analysis (30 cycles × 360 samples per cycle). The median weights among the 30 extractions were computed and the dataset characterized by the highest cosine-similarity (normalized scalar product) with the median weights was chosen as the most representative one. To test the consistency of the median extraction, the mean value of the similarity between the weights of the selected dataset and the remaining 29 extractions was computed for each subject, trial, and synergy. Synergy extraction was applied varying the number of synergies S from 1 to 6 in order to evaluate the coordination complexity. The optimal choice of S was quantified as the smallest number which allowed to reconstruct the EMG envelopes with a total Variance Accounted For (VAF) higher than 90% [11, 22]. VAF values were computed as:

$$ {\mathrm{VAF}}_{\mathrm{S}}=1-\frac{\sum_{\mathrm{i}=1}^9{\sum}_{\mathrm{j}=1}^{10800}{\left({\mathrm{M}}_{\mathrm{i}\mathrm{j}}-\left({\mathrm{W}}_{\mathrm{i}\mathrm{S}}{\mathrm{H}}_{\mathrm{S}\mathrm{j}}\right)\right)}^2}{\sum_{\mathrm{i}=1}^9{\sum}_{\mathrm{j}=1}^{10800}{\left({\mathrm{M}}_{\mathrm{i}\mathrm{j}}\right)}^2} $$

where *i* and *j* respectively indicates one of the nine muscle and a sample of the EMG envelope, while *M*, *W* and *H* respectively indicates the normalized EMG envelopes, the muscle synergies weights, and the activation coefficients.

The total Variance Accounted For by the first synergy (VAF_1_) was used as an additional estimate of synergy complexity [22, 34].

Regardless the inter-subject differences in the number of extracted synergies S, we fixed S to four in order to compare with the behavior of the healthy subjects group. The average set of synergies weights W_HEALTHY_, obtained in a previous study [11], was used for comparison. The four muscle synergies were sorted following the order of healthy muscle synergies by applying cosine-similarity analysis. Each extracted muscle synergy was compared with the healthy weight vectors and the highest cosine-similarity with healthy weights was chosen as criterion to define the synergy order. To identify longitudinal changes in synergy composition, the ordered muscle weights extracted from the patients before and after the intervention were compared with *W*_*HEALTHY*_ by means of the cosine-similarity.

Afterwards, the Nonnegative Matrix Reconstruction (NMR) algorithm [35] was applied, updating iteratively the activation coefficients and fixing the synergies weights for each iteration. The goodness of the reconstruction was assessed computing the VAF values of the NMR. VAF values higher than 0.75 indicate acceptable reconstructions [15]. W_HEALTHY_ was firstly chosen as fixed weights to allow comparisons between stroke and healthy synergy activation profiles [11]. The NMR was then applied to the EMG envelopes at both evaluations (i.e., T1, T2) with the fixed synergy weights of pre-intervention to evaluate longitudinal changes of the activation profiles. The Shape Symmetry Index (SSI) [36] was chosen as the metric to describe the similarity between two reconstructed activation profiles after phase correction:

$$ {\mathrm{SSI}}_{\mathrm{j},\mathrm{l}}=\frac{{\mathrm{C}}_{{\mathrm{h}}_{\mathrm{j},\mathrm{l}}^{\mathrm{T}1}{\mathrm{h}}_{\mathrm{j},\mathrm{l}}^{\mathrm{T}2}}}{\sqrt{\sum_{\mathrm{i}=1}^{360}{\mathrm{h}}_{\mathrm{j},\mathrm{l}}^{\mathrm{T}1}\left(\mathrm{i}\right){\sum}_{\mathrm{i}=1}^{360}{\mathrm{h}}_{\mathrm{j},\mathrm{l}}^{\mathrm{T}2}\left(\mathrm{i}\right)}} $$

where *h*_*j*, *l*_ indicates the reconstructed activation profile of the *j*-th muscle synergy (1 to 4) for the lower limb *l* (left of right), computed by fixing the muscle synergies of the pre-intervention (T1). ${C}_{h_{j,l}^{T1}{h}_{j,l}^{T2}}$ represents the circular cross-correlation function at lag 0. This index could range from − 1 to 1, with higher values indicating more similar shape profile despite possible differences in amplitude.

#### Biomechanical metrics

The biomechanical metrics of the pedaling trials were derived from the force signals acquired in the same 30 cadence-matched pedaling cycles selected for the muscle synergies analysis. Crank angle, tangential and radial force signals were low-pass filtered at 10 Hz with a 3rd order Butterworth filter. As for EMG signals, forces were segmented by pedaling cycle using the synchronized crank angle and then resampled, for each cycle, on a 360-point vector as a function of the crank angle by means of a cubic spline approximation and the left-side profiles were shifted by 180°. The net mechanical work of each leg was computed as the integral of the tangential active force profile, which was derived as the difference between the mean tangential force computed during the active phase and the one obtained during the passive phase [11].

The index of mechanical effectiveness (IE) was used as metric to assess the work dissipation between the voluntary contribution of the tangential force and the total force, computed as the vector sum of the tangential and radial force [11]. Finally, the Area Symmetry Index (ASI) was computed firstly to compare the tangential active force profile of the affected and unaffected leg and then to analyze a single side at once (affected or unaffected) with respect to the mean force profile of the healthy control group [37].

### Statistical analysis

The Wilcoxon signed-rank test was used to compare the pre and post-intervention values for all outcome measures (clinical scales, gait parameters, neuro-mechanical metrics derived both from muscle synergy and force analysis).

For biomechanical metrics (work, IE, ASI) and VAF_1_, the Mann-Whitney U test was used to evaluate differences between patients and healthy subjects [11]. Pearson Chi-squared test for frequencies was used to compare the number of extracted synergies between patients and healthy subjects [11].

The Friedman Test was applied to evaluate the effect of cadence for work, IE, ASI, and VAF_1_. In case of significant changes, a post hoc analysis through a Wilcoxon signed-rank test was performed.

Finally, the Spearman’s correlation coefficient between neuro-mechanical metrics (work and VAF_1_) and clinical outcome measures, including also the gait speed, was computed. All statistical analyses were performed with IBM SPSS Statistics v.25.

## Results

### Participants and clinical changes after treatment

Nine subjects (7 males and 2 females; median [inter-quartile range, IQR] age of 75 [4] years) suffering from a unilateral ischemic stroke in the subacute phase were recruited for this study and completed the 3-week intervention (Table 1). At baseline, all participants were able to walk, but overall, they showed a heterogeneous degree of motor impairment with a gait speed ranging from 35.2 to 118.3 cm/s and the Motricity Index of the affected leg varying from 69 to 100.

**Table 1:** Summary of the participants’ characteristics at baseline

Patient	Gender	Age	Days from stroke	Affected Side	Gait Speed (cm/s)	Motricity Index [0–100]
P1	M	57	14	R	96.3	83
P2	M	74	9	L	118.3	75
P3	M	82	10	R	48.3	75
P4	M	74	18	R	115.5	83
P5	M	67	10	L	81.0	100
P6	F	78	17	L	35.2	75
P7	F	78	30	L	36.4	77
P8	M	75	8	R	77.0	69
P9	M	79	20	R	65.7	75
**Overall**	**7 M – 2 F**	**75 [4]**	**14 [8]**	**5 R – 4 L**	**77.0 [48.0]**	**75 [8]**

Overall values are presented as median [IQR]; *M* Male, *F* Female, *R* Right, *L* Left

After intervention, an overall significant improvement was observed both in terms of clinical scales and gait parameters (Table 2).

**Table 2:** Longitudinal changes in terms of clinical scales and gait parameters

	T1	T2	***P***-value †
**Motricity Index of affected leg [0–100]**	75 [8]	91 [17]	0.031*
**Trunk Control Test [0–100]**	74 [39]	100 [11]	0.031*
**Berg Balance Scale [0–56]**	29 [30]	42 [13]	0.004*
**Functional Independence Measure Motor subscale [13–91]**	46 [22]	65 [21]	0.016*
**Gait speed (cm/s)**	77.0 [48.0]	100.0 [32.0]	0.027*
**Step length of affected leg (cm)**	49.4 [21.9]	53.2 [18.8]	0.129
**Step length of unaffected leg (cm)**	49.0 [12.7]	55.5 [14.1]	0.027*
**Step time of affected leg (s)**	0.70 [0.19]	0.58 [0.05]	0.039*
**Step time of unaffected leg (s)**	0.65 [0.21]	0.57 [0.08]	0.039*
**Double support time of affected leg (s)**	0.50 [0.31]	0.37 [0.11]	0.027*
**Double support time of unaffected leg (s)**	0.49 [0.32]	0.38 [0.11]	0.043*
**Swing velocity of affected leg (cm/s)**	237.45 [131.76]	284.43 [98.68]	0.039*
**Swing velocity of unaffected leg (cm/s)**	235.63 [95.60]	291.53 [82.60]	0.027*

Values are presented as median [IQR]; *T1* Pre-treatment, *T2* Post-treatment. † Wilcoxon signed rank Test. * *p*-values < 0.05

For what concerns the biomechanical metrics, no differences were found in terms of cadence for 11 out of 14 parameters (considering both T1 and T2 evaluation). The only parameters, which showed a significant effect of cadence, were: the work produced by the affected leg (*p* = 0.044) and the ASI between affected and unaffected leg (*p* = 0.012) at T1, and the work of the unaffected leg (*p* = 0.027) at T2. Therefore, biomechanical metrics at 30 RPM were chosen as representative to evaluate longitudinal changes and are reported in Table 3. After training, the work of the affected leg significantly increased (p = 0.027). In terms of cycling efficiency (IE) a wider trend of improvement was found in the affected (17%, *p* = 0.055) than in the unaffected leg (6%, *p* = 0.426), but both presented no significant time effects. Overall, no significant differences with the healthy control group were found before and after training both for works and mechanical efficiency. ASI between affected and unaffected side did not improve over time (*p* = 0.359) and remained significantly lower than ASI between right and left side of the healthy controls (*p* < 0.05) both at T1 and T2. Finally, a significant time effect was obtained for the ASI computed to compare the mean force profile of the healthy controls with the affected (*p* = 0.004) and unaffected leg (*p* = 0.020) of the patients. However, these values resulted both at T1 and T2 significantly lower than normative values (*p* < 0.001).

**Table 3:** Biomechanical metrics evaluated during pedaling trials at 30 RPM

	T1	T2	***p***-value^**†**^	Healthy values [11]	***p***-value (T1 vs H) ^**§**^	***p***-value (T2 vs H) ^**§**^
**Affected Work**	27.76 [25.91]	29.99 [27.61]	0.027*	43.70 [3.52]	0.053	0.074
**Unaffected Work**	30.28 [26.69]	41.50 [26.58]	0.250	43.70 [3.52]	0.102	0.239
**Affected IE**	0.30 [0.18]	0.35 [0.20]	0.055	0.34 [0.06]	0.184	0.732
**Unaffected IE**	0.31 [0.25]	0.29 [0.13]	0.426	0.34 [0.06]	0.569	0.239
**ASI Unaff vs Aff**	0.78 [0.20]	0.83 [0.03]	0.359	0.86 [0.04]	0.014*	0.025*
**ASI Aff vs Healthy**	0.48 [0.25]	0.52 [0.23]	0.004*	0.89 [0.07]	< 0.001*	< 0.001*
**ASI Unaff vs Healthy**	0.52 [0.17]	0.60 [0.19]	0.020*	0.89 [0.07]	< 0.001*	< 0.001*

Values are presented as median [IQR]; *T1* Pre-treatment, *T2* Post-treatment, *H* Healthy group, *Aff* Affected Leg, *Unaff* Unaffected leg, *IE* Index of mechanical Efficiency, *ASI* Area Symmetry Index

† Wilcoxon signed rank Test; § Mann-Whitney U Test; *p < 0.05

### Muscle synergy analysis

The number of pedaling cycles available for muscle synergies analysis ranged between 30 and 103. A high consistency was found among the 30 extractions derived from the 30 normalized EMG envelopes randomly selected among the available ones, for each subject and cadence: the mean similarity between the median extraction, which was used for the following analyses, and the remaining 29 extractions, was higher than 0.85 in 94% of cases, with a total number of cases equal to 288 (4 synergies × 4 cadences × 2 times × 9 patients).

Table 4 reports the number of synergies extracted in all pedaling trials at T1 and T2. At T1, the affected side showed three synergies in the 62% of the trials and the remaining 38% adopted four synergies. The unaffected side presented three and four synergies in the 47 and 50% of trials, respectively, and the remaining 3% had five synergies. After training, the number of synergies did not significantly change in the affected leg (*p* = 0.405), while the unaffected leg showed a significant decrease in the synergies number (*p* = 0.046). Three synergies were found in the 66% of the affected legs, while the 31% and the 5% showed four and five synergies, respectively. The unaffected leg presented three synergies in the 63% and four synergies in the remaining 37%. Overall, the number of synergies extracted from both the affected and the unaffected leg of patients did not significantly differ from the number of synergies characterizing the pedaling of healthy subjects (*p* > 0.05). Concerning VAF_1_, a significant difference between the affected and the unaffected leg was found both at T1 (*p* < 0.001) and T2 (*p* = 0.004), with VAF_1_ of the affected leg being higher. However, no statistically significant changes were obtained over time for neither legs (*p* > 0.05). Overall, the VAF_1_ values were comparable to those estimated from the healthy subjects group, for which a median [IQR] of 0.61 [0.07] was found. A significant difference was found only for the unaffected leg at T1.

**Table 4:** Longitudinal changes in synergy complexity metrics

Synergy complexity	Side	T1	T2	***p***-value† (T1 vs T2)	***p***-value † (T1 aff vs unaff)	***p***-value † (T2 aff vs unaff)	Healthy values [11]	***p***-value (T1 vs H)	***p***-value (T2 vs H)
**N synergies**	Aff	3 [1]	3 [1]	0.405	0.196	0.750	4 [1]	0.132^χ^	0.071^χ^
	Unaff	4 [1]	3 [1]	0.046*				0.590^χ^	0.085^χ^
**VAF**_**1**_	Aff	0.64 [0.08]	0.65 [0.09]	0.185	< 0.001*	0.004*	0.61 [0.08]	0.553^§^	0.919^§^
	Unaff	0.58 [0.07]	0.60 [0.08]	0.385				0.001^§^*	0.054^§^

Values are presented as median [IQR]; *VAF*_*1*_ Variance Accounted For of one synergy, *T1* Pre-intervention, *T2* Post-intervention, *H* Healthy group, *Aff* Affected Leg, *Unaff* Unaffected leg, *H* Healthy Group

† Wilcoxon signed rank Test. χ Pearson’s Chi-squared Test; § Mann-Whitney U Test; *p < 0.05

Synergy complexity metrics was not significantly influenced by the trial cadence in neither leg (*p* > 0.05), except for a significant difference in the number of synergies between 30 and 50 RPM in the unaffected leg at baseline.

#### Correlations with motor impairment at baseline

Table 5 reports the Spearman’s correlation coefficients between age, days post-stroke, clinical scales (MI, BBS, TCT, FIM-motor) and gait speed versus synergy complexity (VAF_1_) and work produced during pedaling by both legs. Significant correlation was found between age and both VAF_1_ (positive correlation) and work (negative correlation) of the affected leg. No significant correlation was found for MI, while moderate to high correlations were found between BBS and TCT and all the neuro-mechanical metrics considered. For what concerns the motor subscale of FIM, correlations were found between VAF_1_ of the unaffected leg and the work of both legs. Finally, the gait speed showed a moderate correlation with synergy complexity of the affected leg and a high correlation with work of both legs.

**Table 5:** Correlations between neuro-mechanical metrics and clinical outcome measures, including gait speed, at baseline

	VAF_**1**_ Affected Leg	VAF_**1**_ Unaffected Leg	Work Affected leg	Work Unaffected Leg
**Age**	0.482	–	−0.807	–
**Days Post-Stroke**	–	–	–	–
**Motricity Index**	–	–	–	–
**Berg Balance Scale**	−0.466	−0.386	0.702	0.745
**Trunk Control Test**	−0.639	− 0.466	0.767	0.800
**Functional Independence Measure Motor subscale**	–	−0.507	0.683	0.667
**Gait Speed**	−0.371	–	0.917	0.900

Only significant Spearman’s correlation coefficients (*p* < 0.05) are reported

Correlation analysis was performed using the trial at 30 RPM

#### Longitudinal changes

**Fig. 2:**
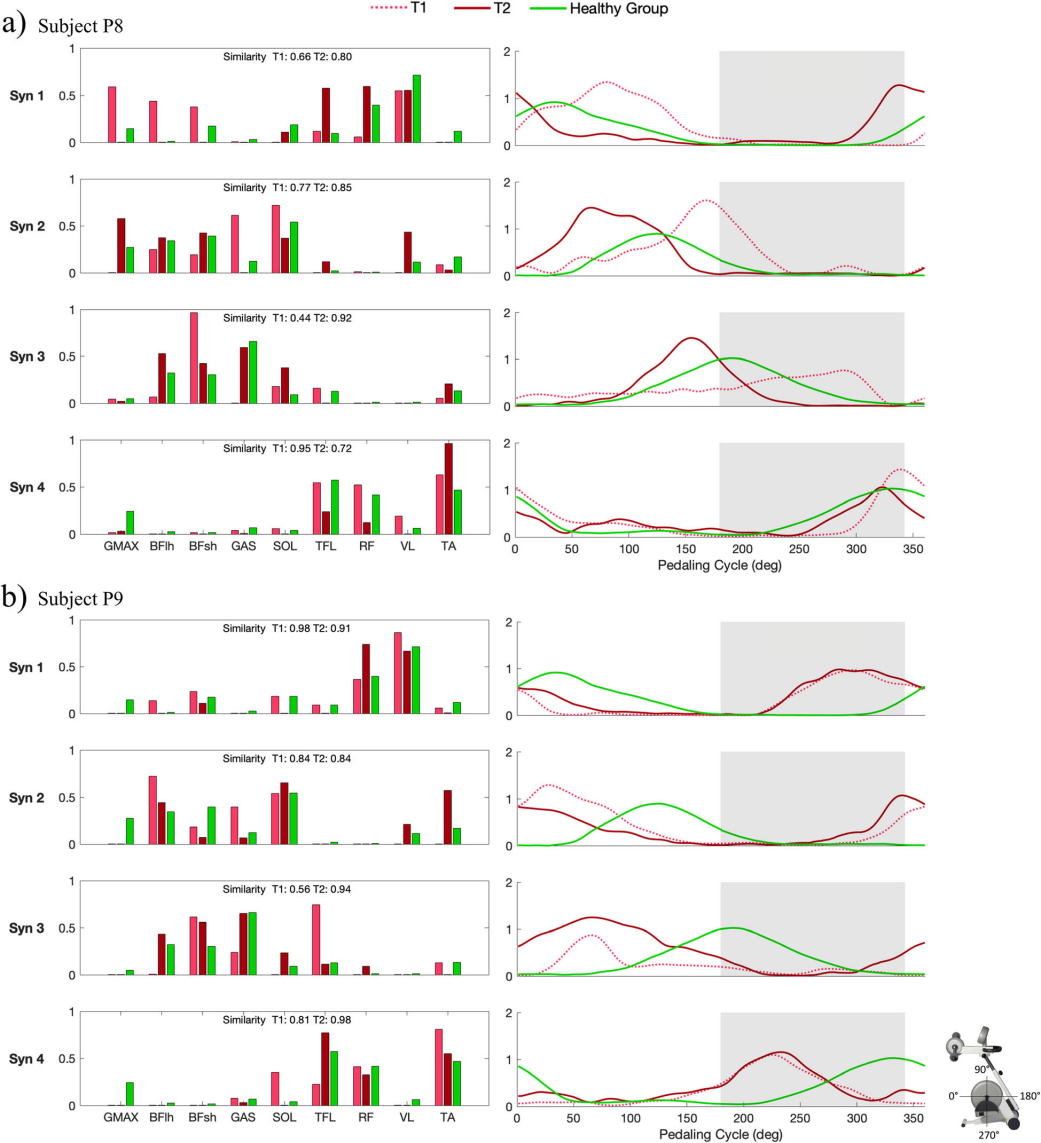
Synergy weights and activation coefficients of the four extracted synergies (Syn1, Syn2, Syn3, Syn4). Muscle synergies extracted before (T1, in light red) and after the training (T2, in dark red) for subject P8 and subject P9 are shown in panel (**a**) and (**b**), respectively. In all panels, the mean synergy weights and activation coefficients obtained by the healthy group [11] are shown in green. In the right panels, the similarity with the healthy weights obtained at T1 and T2 is reported. The left panels show the activation coefficients as function of the pedaling cycle (the grey area represents the knee flexion phase)

Figure 2 shows examples of the affected leg muscle synergies extracted before and after training for two representative subjects, P8 and P9, characterized by a similar walking impairment at T1 but with a different behavior in terms of recovery of walking ability. Subject P8 was characterized by a gait speed of 77.0 cm/s before the intervention; after training he achieved a clinically significant improvement of gait speed, which increased up to 100.0 cm/s. In terms of composition of muscle synergies (Fig. 2-a, left panels), at baseline an alteration in module composition was found, especially for Syn3 (similarity of 0.44), corresponding to the knee flexion phase. After training, the similarity of Syn3 increased up to 0.92; also, the similarity of Syn1 and Syn2 showed an improvement, while for Syn4 there was a decrease from 0.95 to 0.72. Furthermore, the post-treatment improvement in spatial composition corresponded to an improvement in the activation profiles (Fig. 2-a, right panels), which resulted to be more in phase with respect to the physiological ones (in green).

Subject P9 (Fig. 2-b) had a gait speed of 65.7 cm/s at baseline, which decreased to 52.7 cm/s after intervention. In terms of muscle synergies, at baseline he was characterized by a spatial composition similar to healthy subjects but for Syn3, corresponding to the knee flexion phase (similarity of 0.56); after training, an overall improvement of spatial composition was observed, mainly for the most compromised synergy, which achieved a similarity of 0.94. However, differences in terms of activation profiles (Fig. 2-b, right panels) between the patient and the healthy subjects were observed at T1 and no improvements were achieved after training, indicating an overall deficit in the activation timing of “physiological” motor modules.

Figures showing the muscles synergies extracted before and after training for all subjects are included as Additional file 1.

Cosine-similarity analysis of the four extracted synergies did not reveal a significant effect of cadence, except for Syn4 of both legs in post-training assessment (*p* < 0.046); thus, similarity at 30 RPM was reported as representative in Table 6. For the affected leg after intervention, Syn2 and Syn4 showed a slight but not significant improvement over time (*p* > 0.05), while Syn3 significantly increased its similarity with W_HEALTHY_ (median [IQR] change: 0.19 [0.34], *p* = 0.020). No statistically significant changes were observed for the unaffected leg (*p* > 0.05).

**Table 6:** Cosine-similarity of extracted muscle synergies weights of stroke participants at 30 RPM

Side	Synergies	T1	T2	***p***-value †
Affected	Syn1	0.85 [0.23]	0.85 [0.11]	0.820
	Syn2	0.78 [0.07]	0.83 [0.07]	0.129
	Syn3	0.68 [0.30]	0.92 [0.07]	0.020*
	Syn4	0.86 [0.08]	0.96 [0.03]	0.496
Unaffected	Syn1	0.90 [0.12]	0.87 [0.11]	0.570
	Syn2	0.77 [0.05]	0.82 [0.07]	0.496
	Syn3	0.74 [0.42]	0.79 [0.51]	0.734
	Syn4	0.93 [0.07]	0.91 [0.15]	0.203

Values are presented as median [IQR]; *T1* Pre-treatment, *T2* Post-treatment;

† Wilcoxon signed rank Test; *p < 0.05

**Fig. 3:**
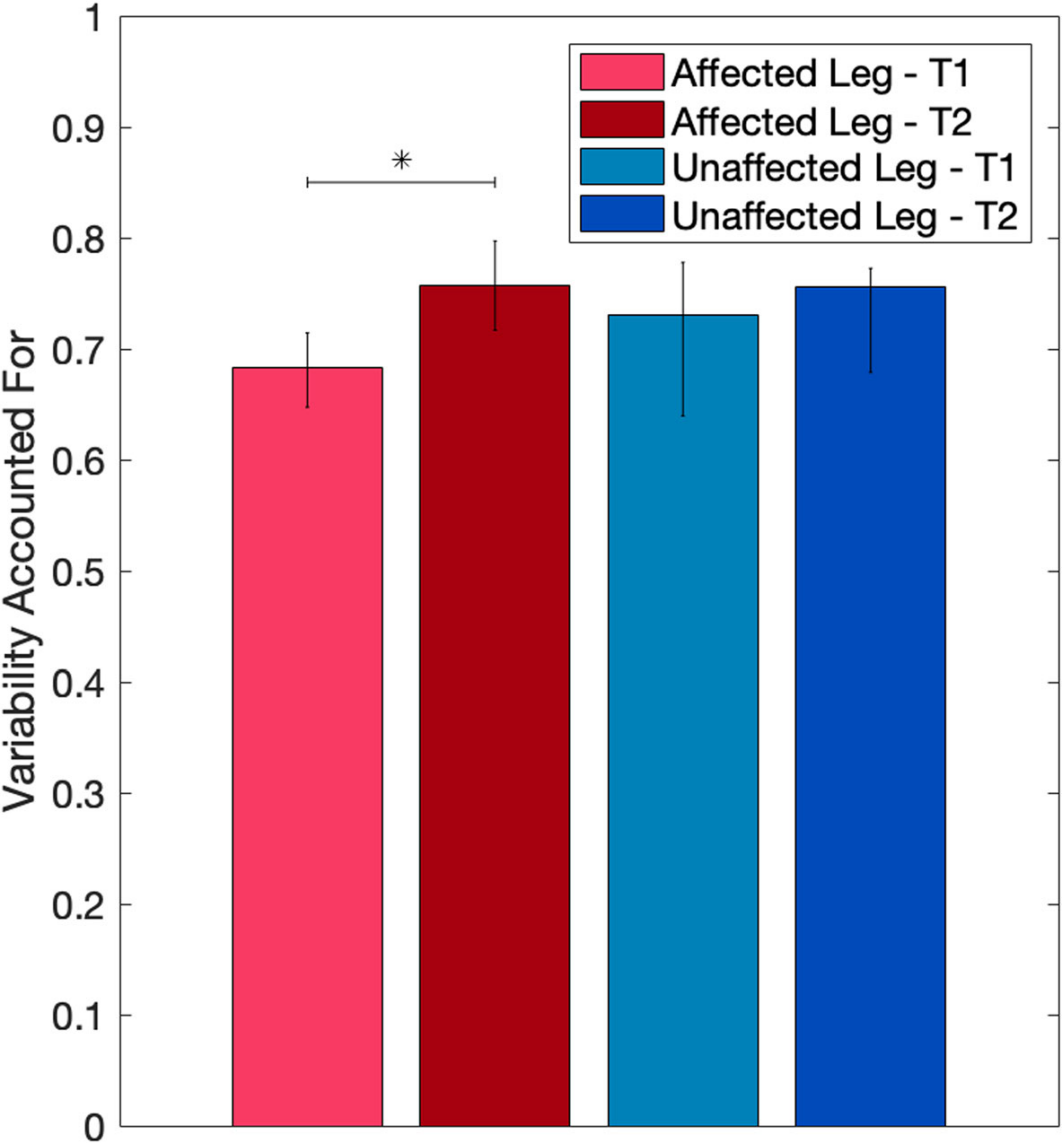
Median [IQR] Variance Accounted For of Non-negative Matrix Reconstruction with *W*_*HEALTHY*_ at 30 RPM. * *p* < 0.05

Muscle synergies weights of the healthy controls was then used to reconstruct the activation timing profiles of the patients’ EMG envelopes. However, before intervention, both the unaffected and the affected legs had a median VAF lower than 0.75 as shown in Fig. 3, and therefore reconstruction was not possible. After intervention, median VAF values slightly overcame the acceptable threshold of 0.75 and significantly increased in the affected leg (*p* = 0.043), while no significant improvements were obtained in the unaffected leg.

The reconstruction of the EMG envelopes post-intervention using the fixed weights obtained pre-intervention was acceptable. Indeed, acceptable VAF values were obtained from the reconstruction of both the unaffected (median [IQR]: 0.77 (0.07)) and the affected leg (0.75 [0.07]).

**Fig. 4:**
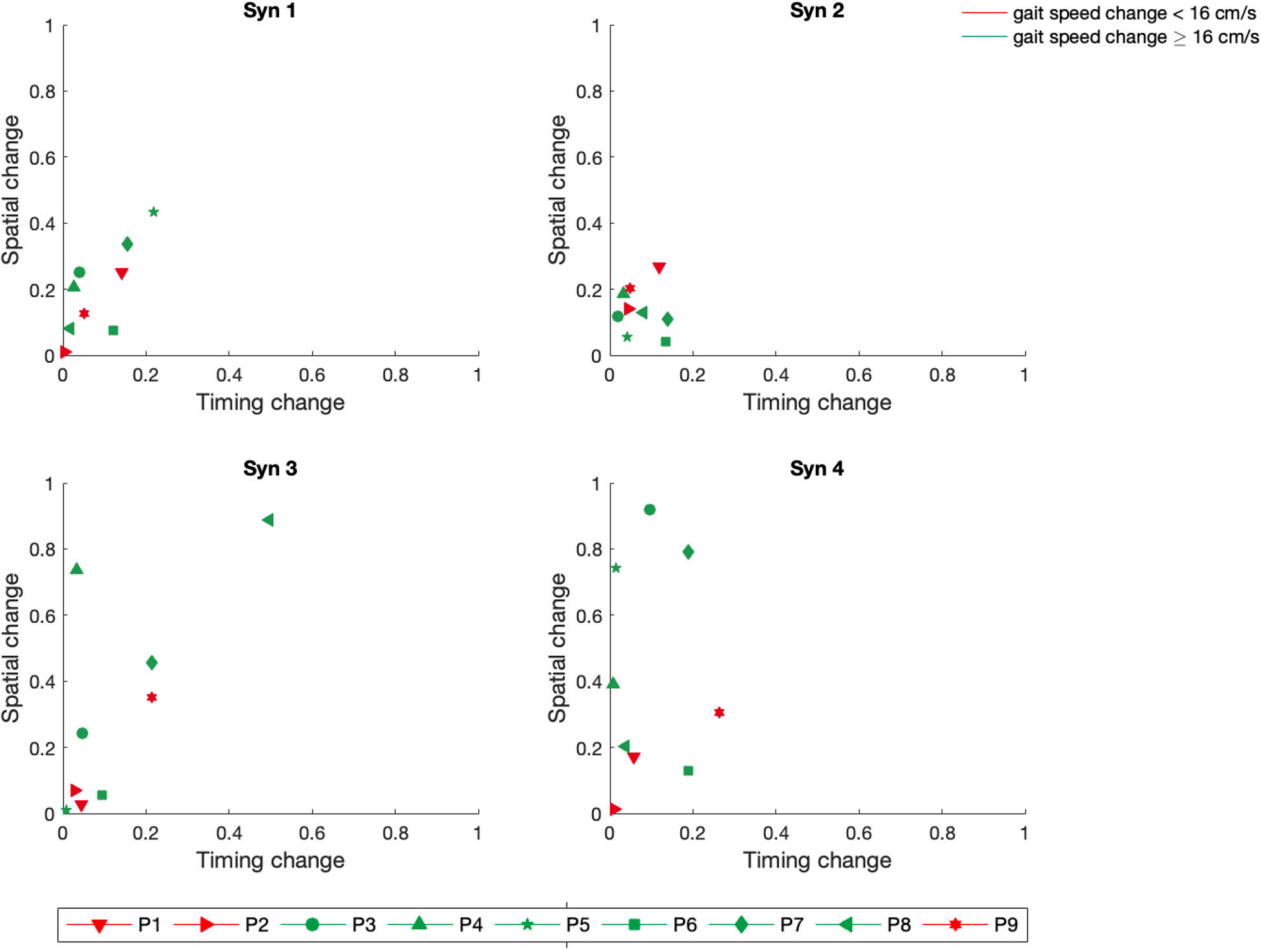
Changes in spatial and timing component of muscle synergies after training. For each muscle synergy obtained by the affected leg, spatial change, i.e. one minus the similarity between the weights extracted at T1 and T2, was plotted against timing change, i.e. one minus the SSI between activation profiles at T1 and T2 after reconstruction with pre-intervention weights. Each patient is reported with a different symbol. Green and red symbols represent patients having a gait speed change after training ≥16 cm/s or < 16 cm/s, respectively

For each patient, the changes in muscle coordination obtained after treatment are displayed in Fig. 4. In each panel, the change in spatial composition is plotted against the change in timing. The change in spatial composition was computed as one minus the similarity between synergies vectors extracted at T1 and T2, while the change in timing was calculated as one minus the SSI between the activation coefficients at T1 and T2 after reconstruction with the pre-intervention weights. When no changes are obtained, both similarity and SSI are equal to 1 and the resulting symbol is placed closed to the origin of the axes. For Syn1 and Syn2, no spatial or timing changes were visible for neither subjects. For what concerns Syn3, 3 out of 6 subjects of the sub-group of patients who clinically improved the gait speed (change ≥16 cm/s [38], in green) showed a consistent change in structure and one of them (P8) also exhibited a change in timing. Finally, for Syn4 4 out of 6 subjects, who clinically improved the gait speed, exhibited higher spatial change than subjects who did not clinically improved the gait speed (in red). No changes in timing was visible for Syn 4.

## Discussion

This study explored muscle synergies analysis during cycling to evaluate the motor recovery process associated with an intervention of 3 weeks of FES-augmented cycling in a group of 9 subacute stroke survivors. Although the clinical outcomes give the best indication of the level of motor impairment of a patient, they are not able to uncover the reasons underneath such condition. Muscle synergies analysis allows a deep investigation about the motor coordination strategy adopted for a specific motor task and it can be used not only to assess patient’s motor impairment but also to investigate the impact of specific interventions on motor coordination changes. In our study the neuro-mechanical analysis of cycling was exploited for both purposes.

The correlation analysis performed at baseline (Table 5) confirmed the good to excellent association between biomechanical metrics and clinical outcomes (Spearman’s coefficient > 0.65) and gait speed (Spearman’s coefficient ≥ 0.9) [11]. These results strongly confirm that cycling represents a valuable task to quantitatively evaluate motor impairments, alternative to walking assessment but inferring information on locomotion ability in the very first phase after stroke.

Previous studies focused on children with cerebral palsy [22, 34] computed the variance accounted for obtained using one synergy (VAF_1_) as summary measure of synergy complexity. The rationale for introducing this outcome measure in the stroke panorama was due to the properties of higher granularity that VAF_1_ has with respect to the number of extracted synergies [22]. In our analysis, the value of VAF_1_ obtained both by the affected and unaffected leg were moderately correlated with motor impairment assessed through balance control and trunk control tests. Whilst only the synergy complexity of the affected side was discreetly associated with gait speed (Spearman’s coefficient = − 0.371, Table 5).

Finally, significant correlations between age and synergy complexity and work of the affected leg were also found (Table 5); these correlations indicated that the increase in age resulted in a decrease of muscle strength of the affected leg (work) and in a decrease of synergy complexity (higher VAF_1_), which might indicate a deterioration of the cortico-spinal drive with age [39].

Synergy composition resulted to be impaired soon after stroke. Indeed, at baseline it was not possible to accurately describe the muscular activation pattern of subacute stroke patients using the same motor modules obtained by healthy controls (reconstruction VAF always lower than 0.75 for both the affected and unaffected legs). A similar result was already obtained by Gizzi and colleagues who investigated the modular coordination of walking in a group of post-acute stroke patients homogeneous in terms of time from stroke (8–20 week), level of impairment (FIM 100–117), and absence of previous rehabilitation [19]. The Authors suggested two possible interpretations of this result: a misdirection of the descending control signals so that different motor modules are activated instead of those usually exploited by healthy subjects to perform the same motor task, and the early occurrence of spinal plasticity. Our sample was recruited very early after stroke (median [IQR]: 14 days [8 days]), and this tends to exclude the hypothesis of spinal plasticity, supporting the hypothesis of a misdirection of the descending signals, which elicit “wrong” sets of modules in the very early phase after stroke, for both the affected and unaffected side. Conversely, this alteration in spatial composition differed from the results obtained in a previous study of our group [11], where the healthy modules were instead able to reconstruct the muscle coordination of both sides of the patients group (mean VAF values > 0.82). This difference might be explained by the more inhomogeneous group, in terms of time after stroke (from 9 to 120 days) and history of rehabilitation, of the previous study with respect to the present study, in which all patients were not involved in previous rehabilitation. This explanation might be also supported by the increase in the reconstruction VAF at the end of the intervention (> 0.75, Fig. 3).

Looking at the longitudinal results, significant changes were obtained after treatment in terms of clinical outcome measures, walking ability (Table 2) and biomechanical metrics of pedaling (Table 3), specifically work of the affected leg and movement symmetry (ASI). However, these changes were not reflected in an increase of synergy complexity, neither in terms of number of synergies extracted nor in terms of VAF_1_. These results confirmed that synergy complexity during cycling did not consistently change with the recovery of function soon after stroke as already observed by Hashigushi et al. when analyzing muscle synergies during walking [24]. Possible explanations could be that in our sample, a number of synergies comparable with that of healthy subjects was obtained by all subjects at baseline, differently from [11], or the limited duration of the training, lasting only 3 weeks. Also, it is worthy to mention that all patients were characterized by a moderate to low level of motor impairment already at baseline, being all able to walk and showing a Motricity Index score ≥ 69/100.

To deeply investigate the influence of motor training on modular coordination, we represented, for each patient, the changes in terms of spatial composition against the changes in activation timing (Fig. 4). The most relevant changes in terms of muscle composition were obtained for subjects characterized by a clinically relevant change in gait speed (≥16 cm/s) after intervention, confirming that changes in the neuro-mechanics of pedaling reflect into improvements of locomotion ability. For what concerns the activation timing instead, no relevant changes were visible (only P8 exhibited a consistent change in timing in Synergy 3). The spatial changes were particularly evident for Synergy 3 and 4, which correspond to the limb recovery phase of the pedaling cycle. The single-subject changes qualitatively observed in Fig. 4 ultimately reflected in a significant group change in the similarity between patients and healthy controls for Synergy 3 of the affected leg (Table 6), suggesting that the changes in muscle composition related to the knee flexion phase were towards a more physiological muscle activation. The knee flexion phase of cycling is recognized as the most compromised after stroke [11, 40] and this was confirmed by our results which showed a median (IQR) value of similarity between patients and healthy controls of 0.68 (0.30) at baseline, as reported in Table 6. A markedly reduction of the peak knee flexion angle of the affected leg is also an abnormality commonly observed in the swing phase of post-stroke gait [41]. This feature associated with a decrease of propulsion and knee flexion velocity at the toe-off contributes to asymmetric gait, increased fear of falling and reduction of walking speed [41]. Therefore, an improvement in the recruitment of Synergy 3 during cycling, corresponding to an improvement in the knee flexion, might translate in an increase of the gait speed and in a more symmetrical locomotion pattern.

During FES-cycling training, the muscles of both legs were stimulated according to a physiological stimulation strategy derived from EMG activations of a group of young healthy subjects so as to provide afferent inputs for the re-learning of the correct motor coordination during pedaling [26]. A first demonstration that FES-cycling training significantly influences motor coordination was already provided by our group using traditional EMG analysis [26]. In this study, we found improvements in the timing of both the biceps femoris and the rectus femoris muscles, which are the main actors of Synergy 3 and 4, respectively. The results obtained in the present study agree with what previously found and further strengthen the potentiality of FES-cycling training in positively influencing neural plasticity soon after stroke.

The analysis of muscle synergies was recently recommended not only to better specify motor impairment, but also to design rehabilitation intervention personalized on the individual impairment [12]. This latter application well suits with FES-based interventions and was already successfully exploited by our group to design a FES controller applied during a gait intervention on a treadmill [7]. The same principle might be applied in the near future to FES-cycling training in order to better personalize the intervention, potentially enhancing the rehabilitative outcomes.

This study presents the following limitations: the sample size of the recruited patients was small (*N* = 9), the patients were not homogeneous in terms of level of motor impairment and we did not include a control group performing only usual care training. Therefore, although promising, our results need to be confirmed on a larger sample size in order to be able to generalize to stroke population. Furthermore, it would be interesting to evaluate modular motor coordination during cycling in a more severely impaired group of patients, not able to walk at baseline. Finally, due to the absence of a control group, this study could not discriminate whether the identified improvements could be directly ascribable to FES-cycling training or were instead related to post-stroke spontaneous recovery as well as to the effects of usual care training. Whether FES-cycling training provides significant changes in muscle synergies beyond that of usual care training and spontaneous recovery remains an open question, which could be answered only by a future randomized controlled trial, including a matched control group.

## Conclusion

Our findings provide supportive evidence that the neuro-mechanical analysis of the cycling movement is a valid assessment of lower limb motor recovery after stroke. Its value in evaluating motor coordination is further enhanced in an early phase after stroke when gait evaluation is not yet possible.

The cycling motor coordination impairment obtained at baseline was due to an altered muscle composition of the muscle synergies, which was particularly evident in the synergy devoted to the pulling phase of cycling. The longitudinal change in clinical scales and gait speed was associated to an increased work of the affected leg during pedaling, in a more symmetrical cycling movement and in a more physiological modular coordination during the pulling phase of the pedaling cycle, which is the most compromised biomechanical function of pedaling after stroke. These results support the role of FES-cycling in influencing motor coordination recovery after stroke but need to be confirmed in a randomized controlled trial with a larger sample size.

## Supplementary information

**Additional file 1.** Muscles synergies extracted for all subjects.

## Data Availability

The data set obtained during the study is available from the corresponding author upon reasonable request.

## Abbreviations

ASI: Area Symmetry Index
EMG: Electromyography
FES: Functional Electrical Stimulation
IE: Index of Effectiveness
IQR: inter-quartile range
NMR: Nonnegative Matrix Reconstruction
RPM: Revolution per minute
SENIAM: Surface Electromyography for Non-Invasive Assessement of Muscles
SSI: Shape Symmetry Index
SYN: Synergy
VAF: Variance Accounted For
WNMF: Weighted Nonnegative Matrix Factorization
