# Supplementary material for: Changes in leg cycling muscle synergies after training augmented by functional electrical stimulation in subacute stroke survivors: a pilot study
Source: J Neuroeng Rehabil. 2020 Feb 27;17:35. doi: 10.1186/s12984-020-00662-w (PMC7047376; doi:10.1186/s12984-020-00662-w)
Supplement: Supplementary file 1 — Additional file 1. Muscles synergies extracted for all subjects. [file 12984_2020_662_MOESM1_ESM.pdf]

Subject P1 (gait speed T1: 96 cm/s , T2: 104 cm/s)

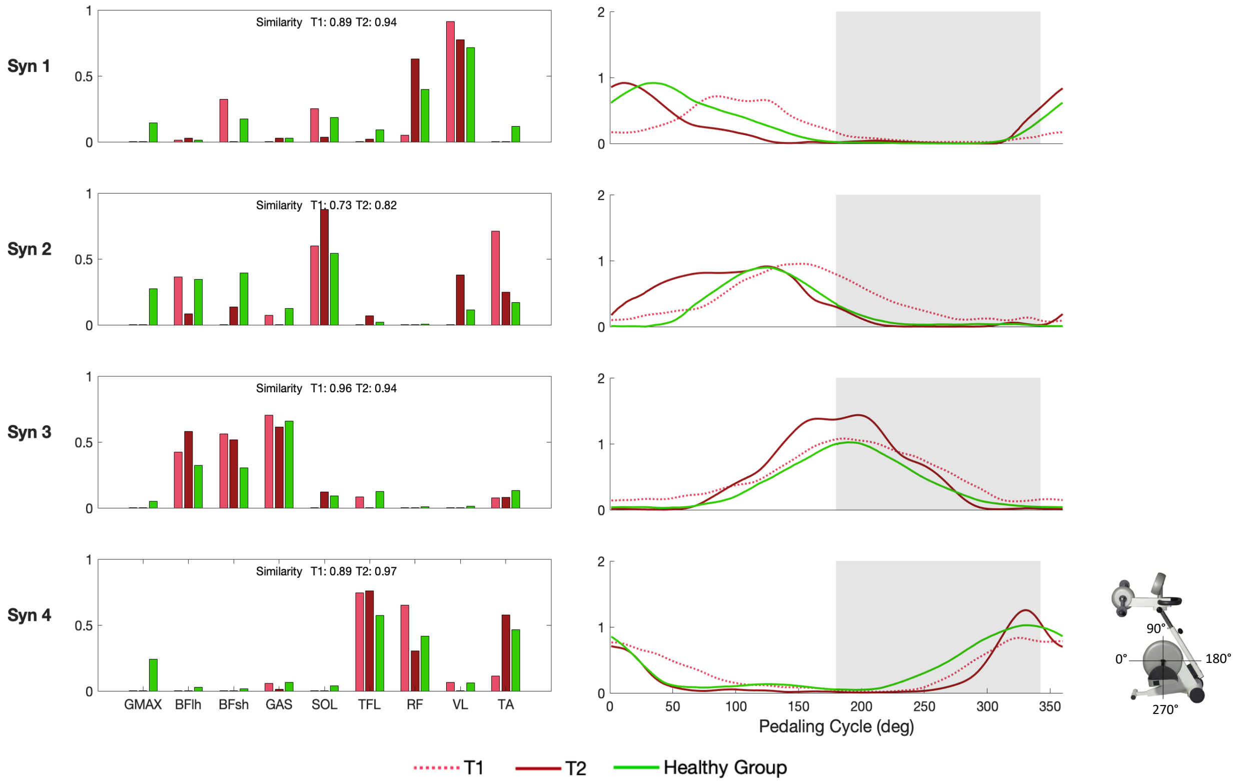

Subject P2 (gait speed T1: 118 cm/s , T2: 116 cm/s)

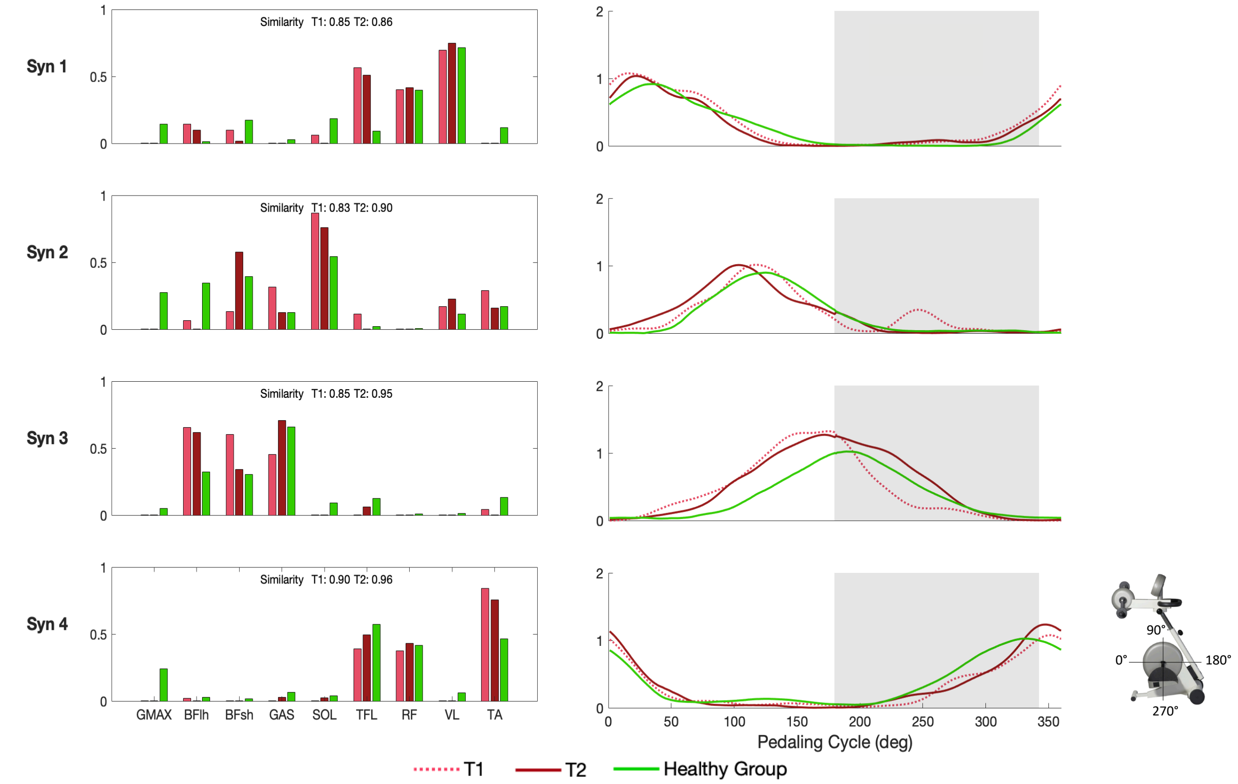

Subject P3 (gait speed T1: 48 cm/s , T2: 72 cm/s)

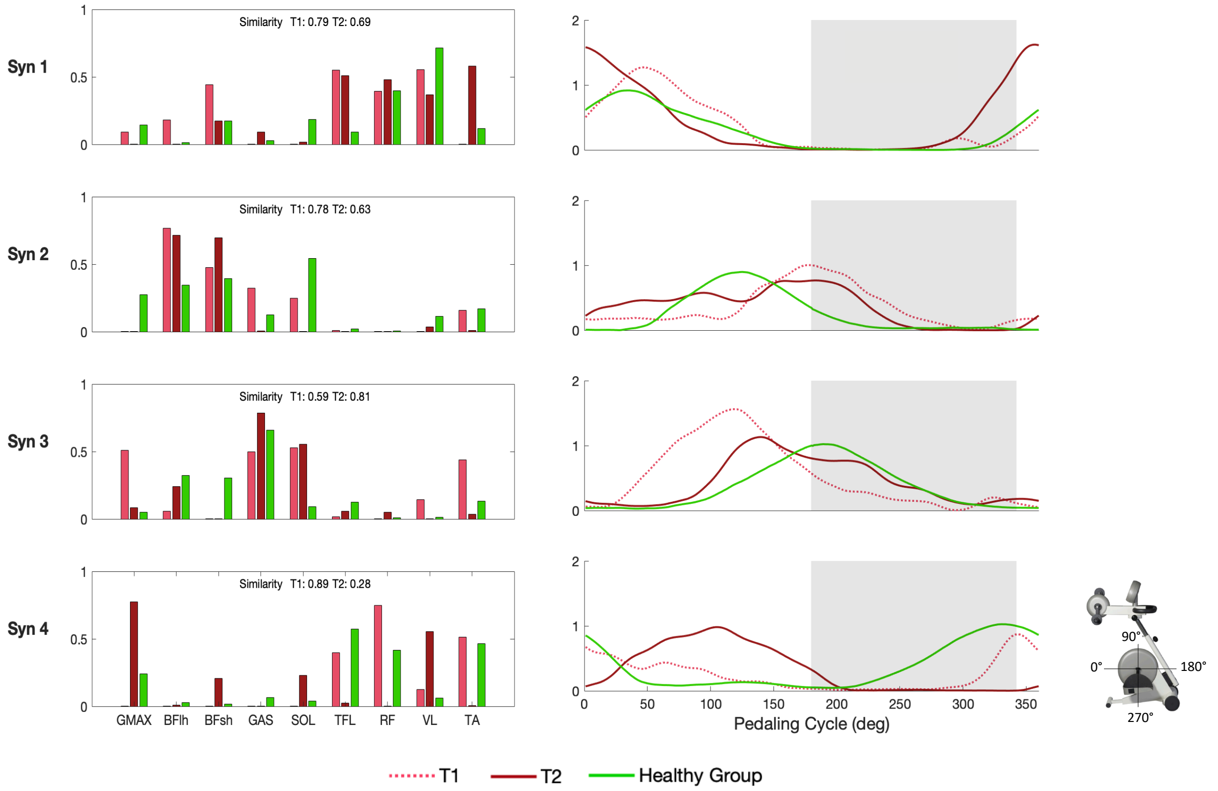

Subject P4 (gait speed T1: 115 cm/s , T2: 164 cm/s)

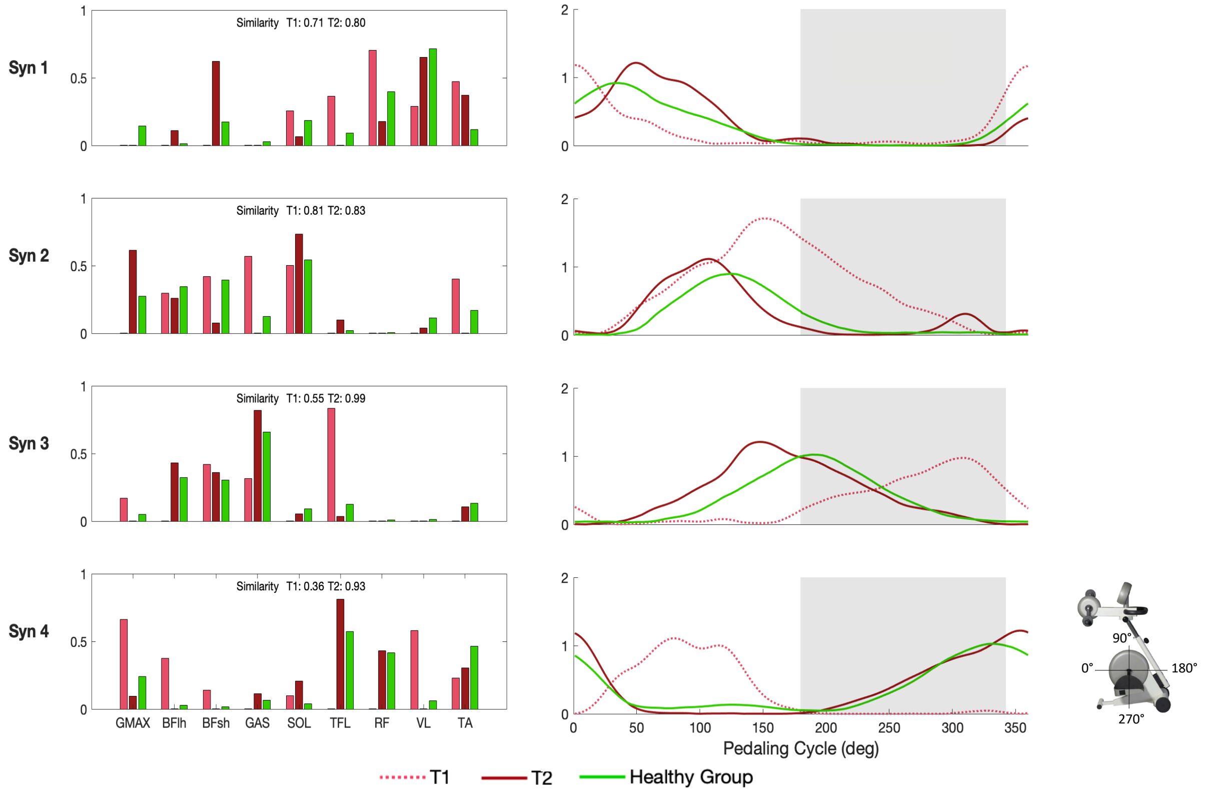

Subject P5 (gait speed T1: 81 cm/s , T2: 100 cm/s)

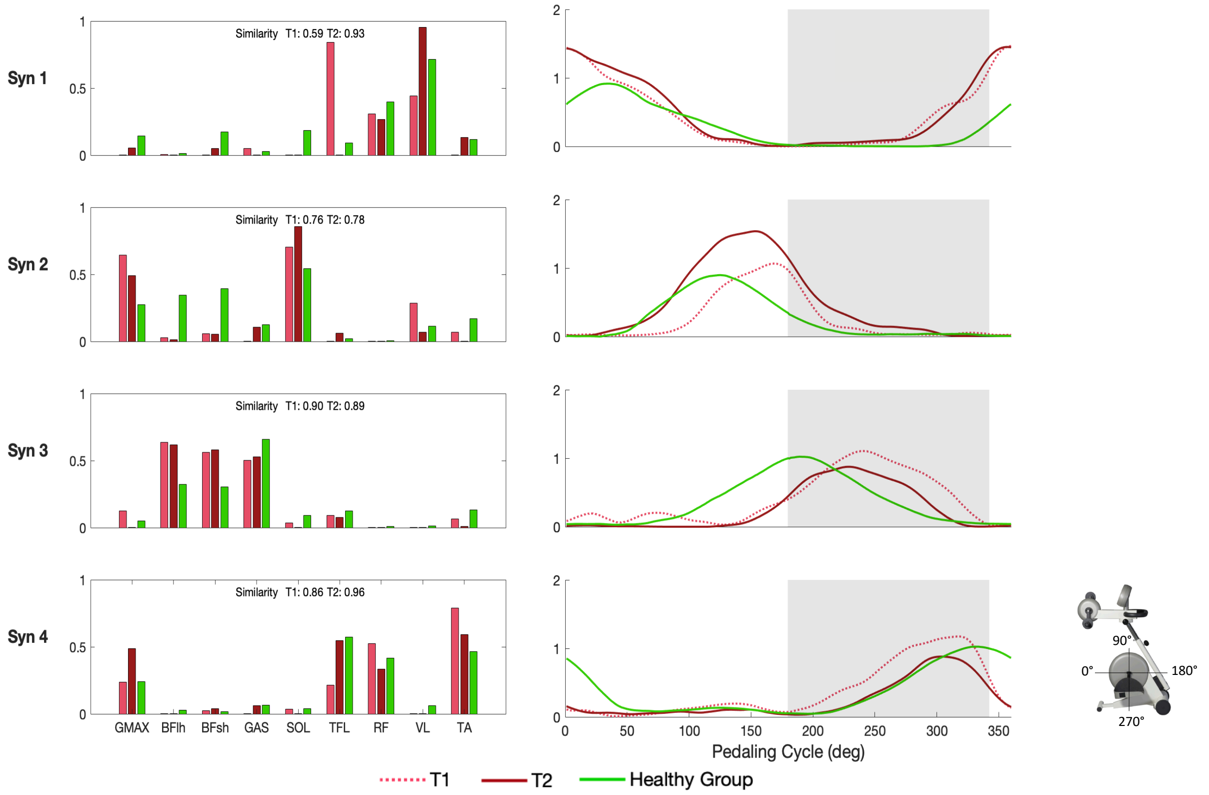

Subject P6 (gait speed T1: 35 cm/s , T2: 56 cm/s)

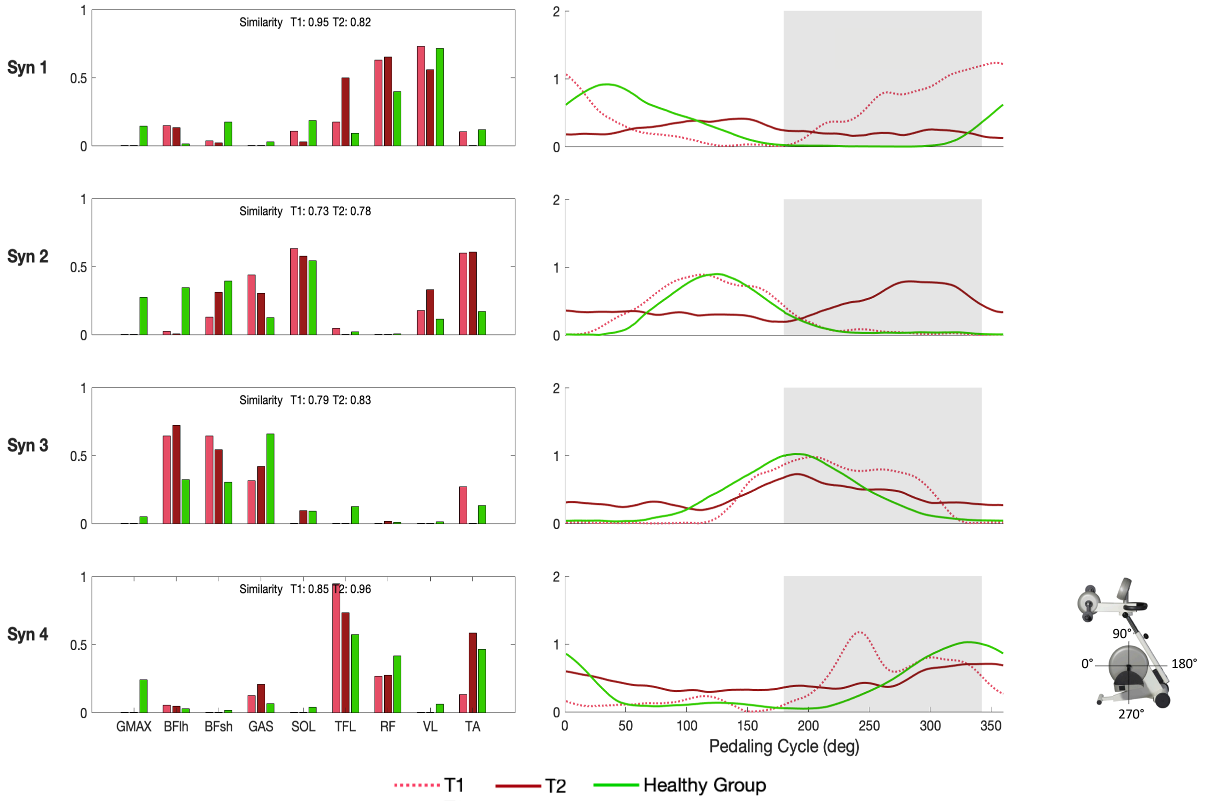

Subject P7 (gait speed T1: 36 cm/s , T2: 74 cm/s)

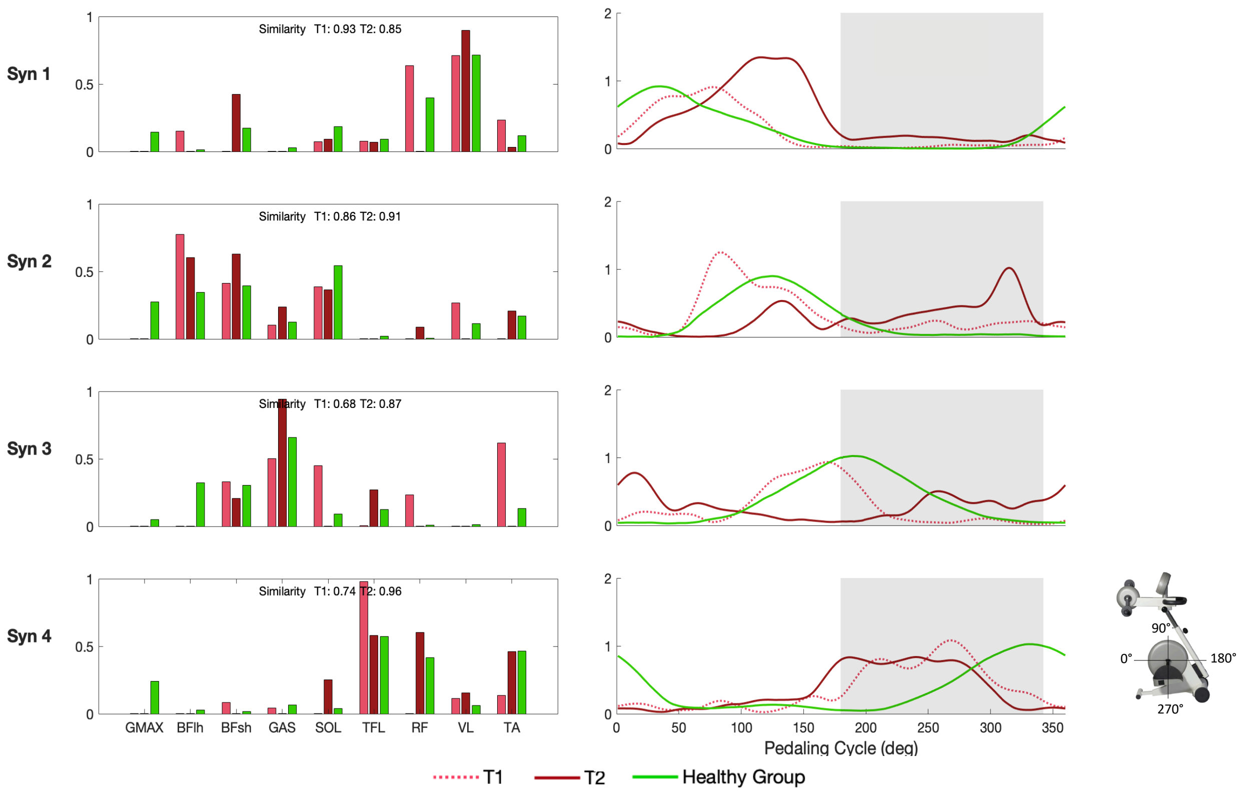

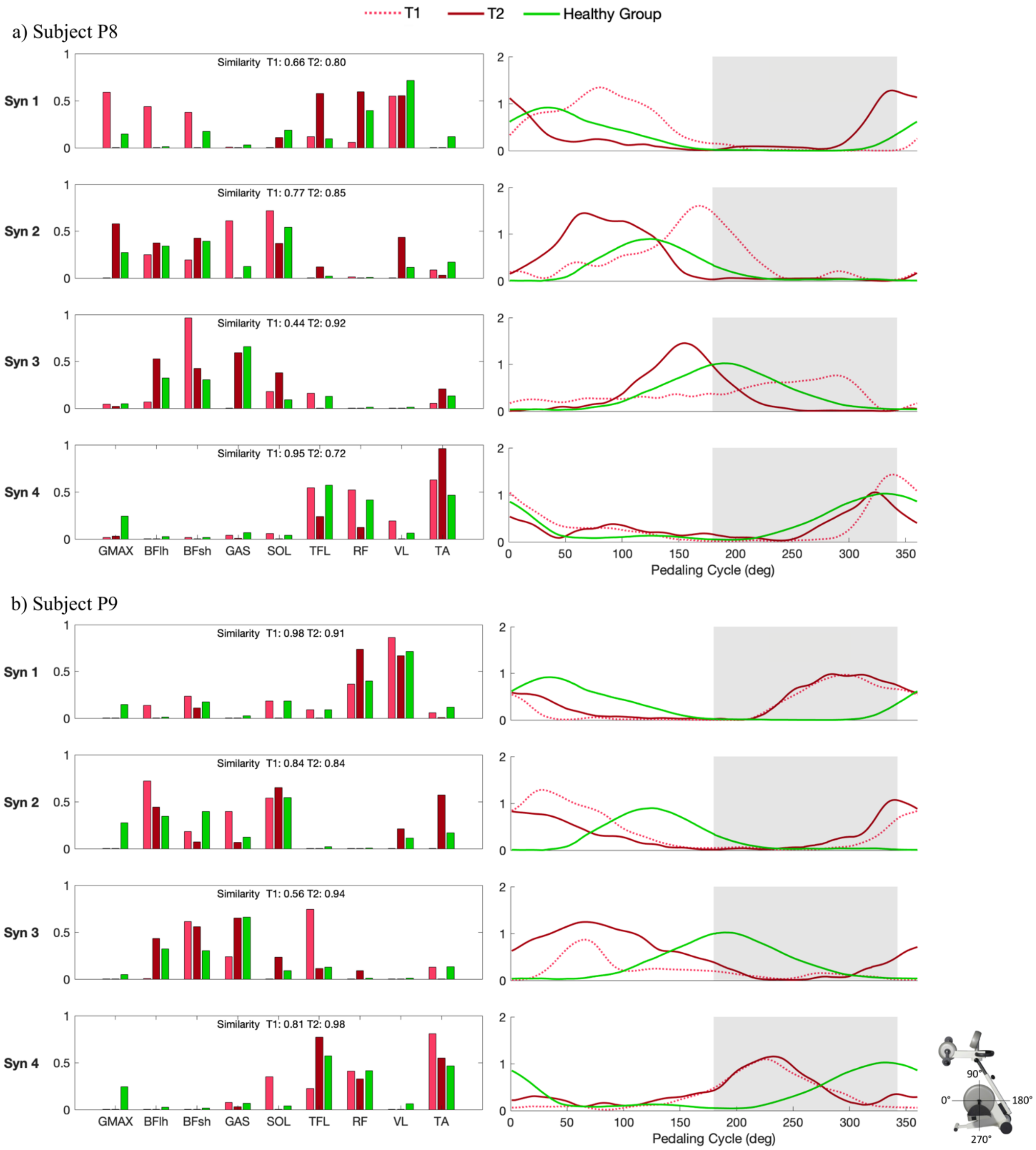

T1
T2
Healthy Group
a) Subject P8
Syn 1
Similarity T1: 0.66 T2: 0.80
Syn 2
Similarity T1: 0.77 T2: 0.85
Syn 3
Similarity T1: 0.44 T2: 0.92
Syn 4
Similarity T1: 0.95 T2: 0.72
GMAX BFlh BFsh GAS SOL TFL RF VL TA
Pedaling Cycle (deg)
b) Subject P9
Syn 1
Similarity T1: 0.98 T2: 0.91
Syn 2
Similarity T1: 0.84 T2: 0.84
Syn 3
Similarity T1: 0.56 T2: 0.94
Syn 4
Similarity T1: 0.81 T2: 0.98
GMAX BFlh BFsh GAS SOL TFL RF VL TA
Pedaling Cycle (deg)
90°
0°
180°
270°
